# The Impact of Diagnostic Imaging on Obstructive Sleep Apnea: Feedback from a Narrative Review

**DOI:** 10.3390/diagnostics15030238

**Published:** 2025-01-21

**Authors:** Salvatore Lavalle, Alberto Caranti, Giannicola Iannella, Annalisa Pace, Mario Lentini, Antonino Maniaci, Ruggero Campisi, Luigi La Via, Caterina Giannitto, Edoardo Masiello, Claudio Vicini, Daniela Messineo

**Affiliations:** 1 Department of Medicine and Surgery, University of Enna Kore, 94100 Enna, Italy; salvatore.lavalle@unikore.it (S.L.); mario.lentini@asp.rg.it (M.L.); 2 Department of Otorhinolaryngology and Audiology, University of Study of Ferrara, 44121 Ferrara, Italy; dott.albertocaranti@gmail.com (A.C.); ruggerocampisi@gmail.com (R.C.); claudio@claudiovicini.com (C.V.); 3 Otorhinolaryngology Department, Sapienza University of Rome, 00042 Rome, Italy; giannicola.iannella@uniroma1.it (G.I.); annalisa.pace@uniroma1.it (A.P.); 4 Surgical Department, Maggiore Hospital, ASP 7, 97100 Ragusa, Italy; 5 Department of Anesthesiology and Intensive Care, Policlinico San Marco, 95123 Catania, Italy; luigilavia7@gmail.com; 6 Department of Diagnostic Radiology, IRCCS Humanitas Research Hospital, 20019 Milan, Italy; caterina.giannitto@gmail.com; 7 Department of Radiology, IRCCS San Raffaele Scientific Institute, 20019 Milan, Italy; masiello.edoardo@hsr.it; 8 Department of Radiological Sciences, Oncology and Anatomo-Pathological Science, “Sapienza” University of Rome, 00184 Rome, Italy; daniela.messineo@uniroma1.it

**Keywords:** Obstructive Sleep Apnea (OSA), diagnostic imaging technologies, artificial intelligence in medicine, airway management, sleep medicine advancement

## Abstract

Obstructive Sleep Apnea is a prevalent sleep disorder characterized by repeated episodes of partial or complete upper airway obstruction during sleep, leading to disrupted sleep and associated comorbidities. Effective, traditional diagnostic methods, such as polysomnography, have limitations in providing comprehensive anatomical detail. Recent advancements in imaging technology have the potential to revolutionize the diagnosis and management of OSA, offering detailed insights into airway anatomy, function, and dynamics. This paper explores the latest innovations in imaging modalities, including high-resolution magnetic resonance imaging, functional MRI, three-dimensional airway reconstructions, and the integration of artificial intelligence algorithms for enhanced image analysis. We discuss the potential of these technologies to improve the precision of OSA diagnosis, tailor treatment strategies, and predict treatment outcomes. Moreover, we examine the challenges of implementing these advanced imaging techniques in clinical practice, such as cost, accessibility, and the need for validation in diverse patient populations. We also consider the ethical implications of widespread imaging, particularly regarding data security and patient privacy. The future of OSA management is poised for transformation as these imaging technologies promise to provide a more nuanced understanding of the disorder and facilitate personalized treatment approaches. This paper calls for continued research and collaboration across disciplines to ensure these innovations lead to improved patient care and outcomes in the field of sleep medicine.

## 1. Introduction

Obstructive Sleep Apnea (OSA) is a prevalent sleep disorder characterized by repeated episodes of complete or partial obstructions of the upper airway during sleep [1]. Heinzer et al. identified a prevalence of OSA, defined by an AHI (Apnea Hypopnea Index) greater than five events per hour, of approximately 49% in men aged 40 to 85 and 24% in women in the same age scale [2]. Moreover, Benjafield et al. described a world prevalence of almost one billion people affected [3]. Therefore, it is plausible that the prevalence of OSAS in the literature is still underestimated [4]. The effort accomplished to restore the airway patency produces fragmentation of sleep and arousal, leading to daytime sleepiness, which is linked to neurocognitive disturbances and to an increased possibility of car accidents [5,6]. The prevalence and severity of OSA have been linked to a variety of adverse health outcomes, including systemic hypertension, cardiovascular disease, stroke, and impaired cognitive function [7,8]. Given the multifactorial nature of OSA, its diagnosis and management require a comprehensive understanding of both the functional dynamics and anatomical structures of the upper airway, as well as the systemic effects of the disorder [9]. Imaging has become an indispensable tool in the diagnosis and therapeutic planning of OSA [10]. Various imaging modalities have been employed to visualize the structures and functions implicated in OSA, each offering unique insights that can guide clinical decision-making. The facial phenotype and craniofacial morphology, which are integral to understanding the risk and pathogenesis of OSA, can be assessed through various imaging techniques, including cephalometry and three-dimensional facial scanning, as reviewed by Agha and Johal [11]. However, traditional radiography, while limited in detail and scope, has been supplemented by more advanced techniques such as magnetic resonance imaging (MRI), computed tomography (CT), and cone-beam computed tomography (CBCT) [12]. Ultrasound, particularly in the form of PoCUS, as reviewed by Burns et al., offers a portable and non-invasive option for assessing the airway and has shown promise in pediatric populations where minimizing radiation exposure is of particular concern [13]. CT and CBCT imaging provide high-resolution images of the bony structures and airway, with CBCT being particularly useful for three-dimensional reconstructions of the airway, as highlighted by Gurgel et al. [14]. These imaging modalities are critical in assessing the airway anatomy for surgical planning or evaluating the effectiveness of oral appliance therapy [15]. MRI stands out for its superior soft tissue contrast, which is invaluable in assessing the pharyngeal anatomy, soft palate, and tongue, as well as the volume of surrounding fat deposits that may influence airway patency [16,17,18]. In addition to these imaging modalities, Whyte and Gibson provide a broader perspective on the utility of imaging in adult OSA, discussing the pathogenesis, diagnostic importance, and the range of imaging tools available, from airway visualization to assessing the neurological and cardiovascular sequelae of the disorder [10,19]. This review aims to integrate the insights from these diverse imaging modalities, providing a comprehensive overview of how each contributes to our understanding of OSA’s pathophysiology, diagnosis, and treatment. By examining the strengths and limitations of each imaging tool, we can better appreciate their roles in the multidisciplinary approach to managing OSA.

## 2. Materials and Methods

### 2.1. Literature Search Strategy

A comprehensive literature search was conducted using multiple electronic databases, including PubMed, Scopus, Web of Science, and Google Scholar. The literature search included studies from 1990 to 2024. The search aimed to identify peer-reviewed articles that discussed the use of imaging modalities in diagnosing, evaluating, and managing OSA. Keywords and phrases used in the search included “obstructive sleep apnea”, “imaging”, “MRI”, “CT”, “CBCT”, “ultrasound”, “echocardiography”, “cephalometry”, “facial phenotype”, and “airway assessment”. Boolean operators (AND, OR) were used to refine the search results.

### 2.2. Selection Criteria

**Figure 1:**
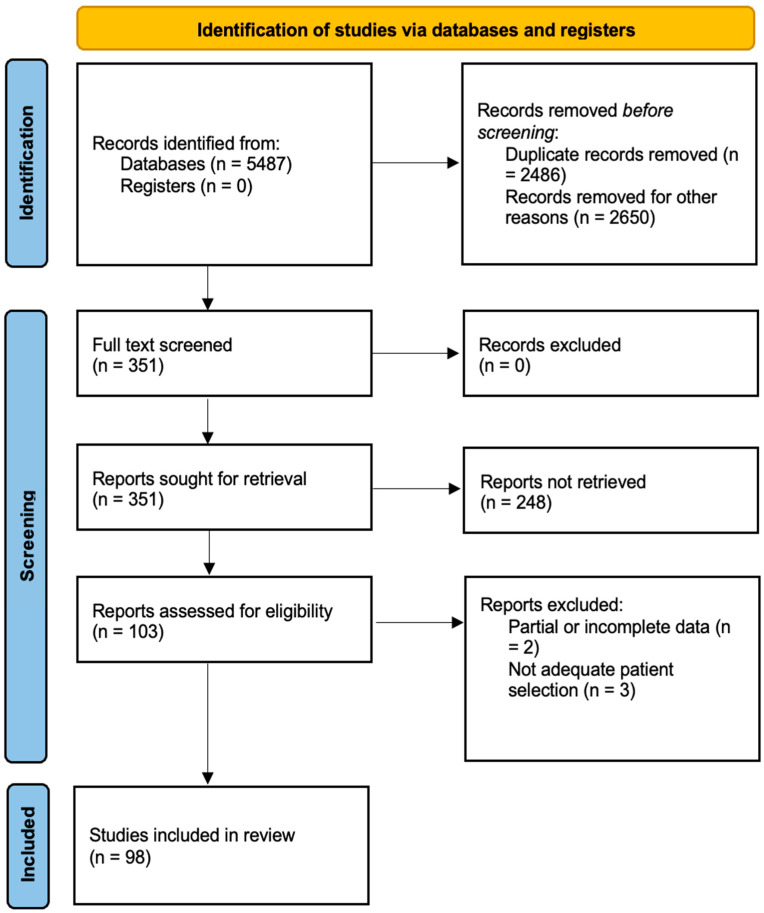
PRISMA flow diagram describing the literature research protocol. PubMed, SCOPUS, Web of Science, and Google Scholar.

The studies were selected based on the following inclusion criteria: articles published in English, studies that specifically addressed the use of imaging tools in OSA, research including human subjects with a diagnosis of OSA, articles that provided clear methodological details and reported on imaging outcomes, and studies that included both adult and pediatric populations. The exclusion criteria were studies that did not focus on imaging modalities, case reports, editorials, and commentaries, and research with inconclusive or incomplete data. From an initial review of 5487 abstracts, 5136 were excluded based on relevance and adherence to the inclusion criteria. A total of 351 full-text articles were assessed, of which 98 were ultimately included in this review (Figure 1).

### 2.3. Data Extraction and Synthesis

Two independent reviewers (A.C. and A.M.) extracted data from the selected studies, with any discrepancies resolved through discussion or by consulting a third reviewer (C.V.). The extracted data included author names, year of publication, study design, population characteristics, imaging modalities used, key findings, and conclusions. The data were synthesized to provide a narrative review of the imaging modalities used in OSA. The synthesis involved a comparative analysis of the different imaging tools, their applications in OSA, and the insights they provided into the disease’s pathophysiology, diagnosis, and treatment. We adopted the AMSTAR guidelines to the data synthesis, retrieving and elaborating all the information from the literature regarding imaging application in OSAS to formulate specific questions and responses [20].

### 2.4. Quality Assessment

The quality of the included studies was assessed using standardized checklists appropriate for each study design. The Cochrane Collaboration’s tool for assessing the risk of bias was used for randomized controlled trials. For observational studies, the Newcastle–Ottawa Scale was employed. The quality assessment helped to ensure that the conclusions drawn from this review were based on high-quality evidence.

### 2.5. Ethical Considerations

As this review did not involve the collection of primary data from human subjects, ethical approval was not required. However, this review was conducted following the ethical standards laid out in the 1964 Declaration of Helsinki and its later amendments.

## 3. Results and Discussion

### 3.1. What Is the Role of Imaging in the Initial Diagnosis of OSA, and How Do Different Modalities Compare in Their Diagnostic Capabilities?

**Figure 2:**
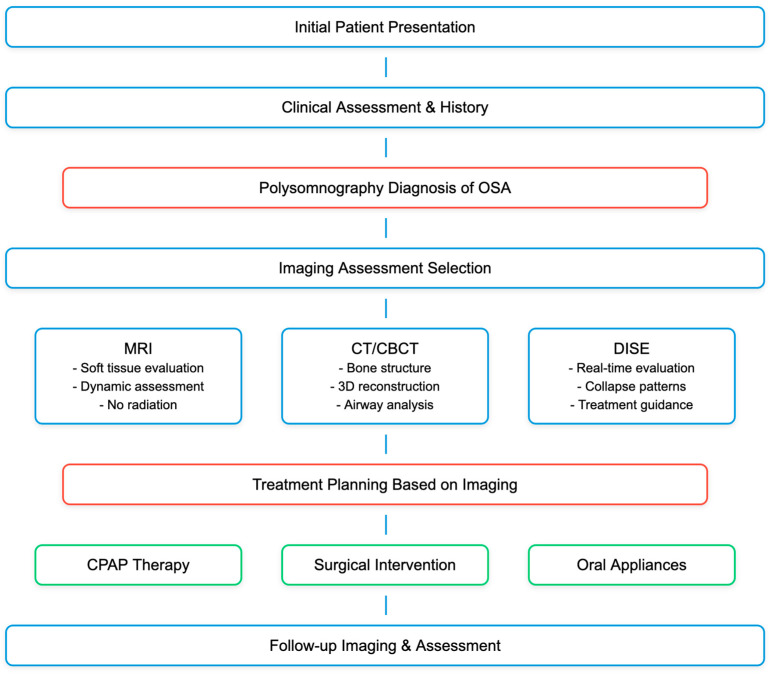
Radiologic management of patients with OSA.

Imaging plays a critical role in the initial diagnosis and management of OSA, a condition characterized by repeated episodes of partial or complete obstruction of the upper airway during sleep [1,10]. These obstructions can lead to fragmented sleep, reduced oxygen saturation, and subsequent daytime sleepiness, as well as long-term cardiovascular risks [21]. The initial diagnosis of OSA typically begins with a clinical evaluation and patient history, followed by a polysomnography (PSG) test to monitor sleep, breathing patterns, and oxygen levels [22]. However, PSG does not provide information about the anatomical causes of the airway obstruction [23]. This is where imaging techniques become essential (Figure 1). Different imaging modalities offer varying advantages. MRI provides excellent soft tissue contrast and can delineate structures within the airway without ionizing radiation. It is particularly useful for visualizing the entire airway and surrounding tissues and can be used to assess changes in airway caliber during different phases of respiration [12]. Conversely, CT scans offer detailed bone and soft tissue imaging and are faster than MRI, which can be beneficial for claustrophobic patients [24,25]. However, they involve ionizing radiation, which is a consideration when imaging is required repeatedly or in vulnerable populations such as children. Contrarily, X-rays are less commonly used due to their limited detail but can offer a quick overview of the airway structure. They are of limited use in soft tissue evaluation but can be useful in assessing bony structures and large tissue enlargement [26] (Figure 2). The literature consistently supports the integration of imaging modalities in cases where understanding the structural causes of airway obstruction is essential, particularly when planning surgical or other interventional treatments. These imaging techniques, though they do not replace PSG, enhance the diagnostic pathway by offering insights into the pathophysiology of OSA and enabling tailored treatment approaches [27].

### 3.2. How Does Imaging Assist in the Treatment Planning for OSA, and What Are the Implications for Patient Outcomes?

Imaging is a critical component of treatment planning for OSA as it provides a visual representation of the airway that can guide therapeutic decisions. Imaging can pinpoint the level(s) of airway obstruction, which is crucial for selecting the appropriate intervention, whether it be CPAP, oral appliances, or surgery [19]. For patients undergoing surgery, preoperative imaging is used to plan the surgical approach [16]. For example, the size and shape of the jawbone assessed by CT scans can influence the type of maxillomandibular advancement performed [28]. Imaging can guide the design of oral appliances by showing the relationship between the mandible, tongue, and airway, allowing for a more effective and comfortable fit [29,30]. In addition, post-treatment imaging can assess changes in airway size and structure, providing feedback on the effectiveness of the treatment, monitoring treatment efficacy [16]. Table 1 summarizes the various imaging procedures commonly used for the evaluation of OSA.

### 3.3. What Are the Specific Applications of Drug-Induced Sleep Endoscopy (DISE) in the Management of OSA, and How Does It Compare to Other Imaging Techniques?

DISE is a unique imaging modality that offers real-time visualization of the airway during a state of induced sleep initially introduced by Croft and Pringle in 1989 and further developed during the 1990s [39,40]. The technique involves evaluating the upper aerodigestive tract using a flexible endoscope while the patient is pharmacologically induced into a sleep-like state that emulates physiological sleep experienced during the night in order to assess the anatomy of these regions during sleep. The instrument is passed through the nostrils to examine the nasal cavities, nasopharynx, oropharynx, hypopharynx, larynx, and sometimes the trachea, aiming to identify the obstructive site underlying OSAS [41]. Over the years, the procedure has demonstrated to be safe for the patient, repeatable, and reliable from an inter-observer perspective [42]. Its specific applications in OSA management include identifying the site of airway collapse. DISE allows for a dynamic assessment of the airway, providing insight into the level and pattern of obstruction, which static imaging cannot capture [43]. The detailed view of airway obstruction during DISE can inform the surgical strategy, such as the type of tissue that needs to be removed or repositioned during procedures like UPPP (uvulopalatopharyngoplasty) or MMA (maxillomandibular advancement), tailoring surgical interventions [43,44]. Furthermore, for patients who are intolerant to CPAP, DISE can help in evaluating the potential success of alternative treatments like oral appliances or positional therapy by observing the airway’s response to these interventions [40].

### 3.4. How Does Imaging Influence the Selection of Surgical Candidates Among OSA Patients, and What Factors Are Considered to Optimize Patient Selection and Surgical Outcomes?

Imaging is a critical tool in the selection of surgical candidates for OSA treatment. It provides detailed anatomical information that can help predict which patients are likely to benefit from surgery and which specific surgical procedures may be most effective. Imaging can reveal the size, shape, and position of the jaw, tongue, tonsils, and other structures that may contribute to airway obstruction. This helps in identifying patients whose anatomy is amenable to surgical correction [12,45]. Techniques like CT scans and MRI can help visualize the entire airway in three dimensions, which is essential for planning surgeries such as maxillomandibular advancement or tracheostomy [46,47]. Moreover, some advanced imaging techniques allow for virtual surgery simulations to predict the outcome of certain surgical procedures, thus aiding in the decision-making process [48,49].

### 3.5. What Are the Advancements in Imaging Techniques That Have Improved the Understanding of OSA Pathophysiology, and How Have These Advancements Impacted Clinical Practice?

Advancements in imaging techniques have substantially augmented our comprehension of the pathophysiology underlying OSA. These advancements encompass a range of modalities with different levels of complexity and information provided, each contributing unique insights into the structural and functional aspects of the disorder (Figure 2). Among the innovative techniques available, Dynamic Imaging Techniques like DISE provide a dynamic assessment of the airway during induced sleep, offering insights into the functional aspects of airway collapse that static imaging cannot provide [40,43]. In contrast, 3D Reconstruction and Computational Modeling allow for a more comprehensive analysis of the airway structure and function, including the simulation of airflow and tissue mechanics, which can help in understanding the individual pathophysiology of OSA [37,49,50]. In addition, Functional MRI (fMRI) can measure changes in blood flow related to neural activity in response to airway obstruction during sleep, providing insights into the central nervous system’s role in OSA [51]. All these advancements have impacted clinical practice by improving diagnostic accuracy and enhancing imaging techniques, which has led to more accurate diagnoses by allowing clinicians to see both the static and dynamic aspects of the airway. With a better understanding of the pathophysiology of OSA in individual patients, clinicians can tailor treatments more effectively, consenting a personalized treatment. Moreover, advanced imaging has improved the precision of surgical interventions, potentially leading to better outcomes, enhancing surgical planning. Despite these advancements, it is essential to consider the geographic variability in the approach to OSAS patients. For instance, as highlighted in the study by Neagos et al., differences in healthcare access, demographic characteristics, and cultural factors significantly influence how OSAS is diagnosed and managed in different regions. This variability underscores the need for region-specific strategies to optimize patient care and treatment outcomes [52].

### 3.6. Can Imaging Modalities Predict Long-Term Compliance and Success with CPAP Therapy, and What Are the Implications for Patient Management and Follow-Up Care?

Imaging modalities have the potential to predict long-term compliance and success with CPAP therapy to some extent. Imaging can reveal structural changes in the upper airway that may correlate with CPAP efficacy [53]. For instance, a larger tongue or a smaller lateral pharyngeal wall area might predict a lower likelihood of CPAP compliance or success [54]. Post-treatment imaging can show changes in airway size or shape with CPAP use, which may be indicative of treatment success and could potentially predict long-term compliance [16,55,56,57]. The implications for patient management and follow-up care include customizing CPAP therapy. Imaging findings can help in adjusting CPAP settings or in the selection of CPAP mask types for individual patients [33,54]. For patients likely to be non-compliant with CPAP, imaging can guide the selection of alternative treatments, such as oral appliances or surgery [31,58]. Regular imaging can be used to monitor the effectiveness of CPAP therapy and adjust as needed [59,60,61].

### 3.7. What Are the Potential Risks and Limitations Associated with the Use of Imaging in the Diagnosis and Management of OSA, and How Can They Be Mitigated?

**Figure 3:**
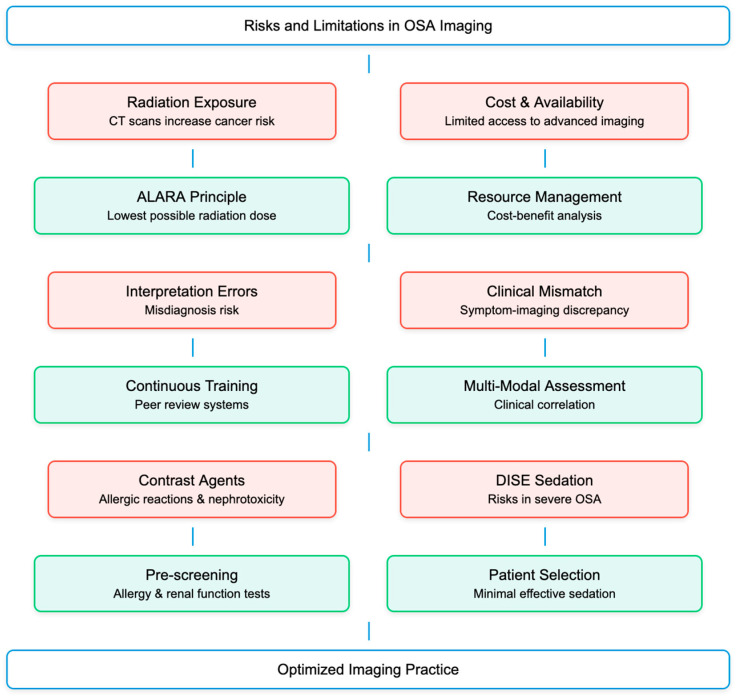
Flow diagram of potential risks and limitations related.

While imaging is a valuable tool in the diagnosis and management of OSA, there are potential risks and limitations to consider. Modalities such as CT scans expose patients to ionizing radiation, which can increase the risk of cancer over time [62]. To mitigate this, clinicians should adhere to the “as low as reasonably achievable” (ALARA) principle, using the lowest possible radiation dose to achieve the necessary diagnostic information [63,64]. Advanced imaging techniques can be expensive and not readily available in all healthcare settings. Cost–benefit analyses and consideration of healthcare resources are essential to ensure that imaging is used judiciously. Imaging requires an accurate interpretation by experienced radiologists or sleep specialists. Misinterpretation can lead to incorrect diagnoses or inappropriate treatment plans. Continuous training and peer review can help reduce the risk of errors. Imaging may sometimes suggest the presence or absence of an abnormality that does not correlate with the patient’s clinical symptoms, leading to unnecessary treatments or missed diagnoses. Correlating imaging findings with clinical evaluation and other diagnostic tests can help minimize these issues [25,65,66,67]. Some imaging studies require contrast agents, which can cause allergic reactions or nephrotoxicity [68,69]. Pre-screening for allergies and renal function assessment can help mitigate these risks. Procedures like DISE require sedation, which carries its own risks, particularly in patients with severe OSA [70,71]. Proper patient selection, monitoring, and the use of the lowest effective sedation dose can help reduce these risks (Figure 3).

### 3.8. How Do Advancements in Machine Learning and Artificial Intelligence Contribute to the Analysis of Imaging Data in OSA, and What Are the Potential Benefits and Challenges?

**Figure 4:**
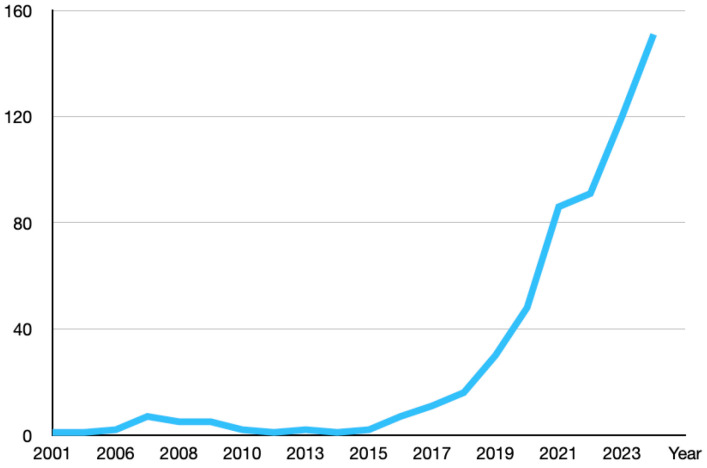
Trend of publications per year on PubMed related to machine learning and AI-based analysis of OSA. The graph demonstrates a notable increase in research activity over recent years, reflecting the growing interest and advancements in applying artificial intelligence to the field of sleep medicine.

In recent years, there has been a significant increase in the number of publications focusing on machine learning and AI-based analysis for OSA (Figure 4). AI algorithms can quickly analyze large volumes of imaging data, identifying patterns and anomalies that may be indicative of OSA, which can improve diagnostic speed and accuracy [72]. Machine learning models, particularly supervised learning algorithms, have been employed to analyze complex datasets derived from imaging modalities. Convolutional neural networks (CNNs) are extensively utilized for image classification and feature extraction, enabling the identification of anatomical structures associated with OSA. For instance, CNNs have been applied to automate the detection of craniofacial abnormalities in cephalometric radiographs, facilitating early diagnosis [73]. Support vector machines and random forests have also been applied to imaging data, such as brain diffusion tensor imaging, to differentiate OSA patients from healthy controls. These models analyze subtle variations in brain structure that may be indicative of OSA, contributing to a more comprehensive understanding of the disorder’s impact on neural anatomy [74]. Additionally, hybrid models combining CNNs with Transformer architectures have been developed to detect apnea–hypopnea events using radar-based imaging, offering a non-intrusive diagnostic alternative. These models leverage the strengths of both architectures to improve detection accuracy and provide real-time analysis [75]. Generative adversarial networks (GANs) have been explored to enhance image quality and generate synthetic imaging data, addressing challenges posed by limited datasets and improving the robustness of AI models in OSA diagnosis. By generating high-fidelity synthetic images, GANs can augment training datasets, enabling models to learn from a more diverse array of scenarios [76]. AI can help in developing predictive models for treatment outcomes, potentially identifying which patients are likely to respond well to certain therapies [77,78,79]. By analyzing imaging data in conjunction with other patient data, AI can contribute to more personalized treatment plans tailored to the individual characteristics of a patient’s OSA [80]. However, there are challenges associated with AI in imaging. AI algorithms require large, high-quality datasets to be trained effectively. Inconsistent or poor-quality imaging data can lead to inaccurate AI models. AI decisions can sometimes be a “black box”, making it difficult for clinicians to understand how the algorithm arrived at a conclusion. Efforts are being made to develop more interpretable AI systems [81,82]. Integrating AI tools into existing healthcare systems and workflows can be challenging and requires careful planning and training [83]. There are concerns about patient privacy, data security, and the ethical implications of AI in healthcare. AI systems often require access to vast amounts of personal health information to function effectively, raising the risk of data breaches and unauthorized access. Ensuring the confidentiality and integrity of patient data is paramount to maintain trust in healthcare systems. Ethical considerations also emerge, particularly related to informed consent, data ownership, and the potential for algorithmic bias. Patients must be adequately informed about how their data are utilized by AI systems, and safeguards should be in place to prevent biases that could lead to unequal treatment outcomes. Robust regulatory frameworks and ethical guidelines are needed to address these issues [84,85,86]. In the United States, the Health Insurance Portability and Accountability Act (HIPAA) sets standards for protecting sensitive patient information. Similarly, the European Union’s General Data Protection Regulation (GDPR) enforces strict data protection and privacy laws.

### 3.9. What Is the ROLE of Polysomnography in Conjunction with Imaging Studies in the Comprehensive Evaluation of OSA, and How Do These Tools Complement Each Other?

Polysomnography is the gold standard for diagnosing OSA and involves an overnight study that records various physiological parameters during sleep [87]. When used in conjunction with imaging studies, these tools provide a comprehensive evaluation of OSA. The PSG contains fundamental information for evaluating patients with OSAS. In addition to the AHI, data concerning apneas, hypopneas, snoring data, and oxygen saturation data (such as time spent below 90% saturation, the so-called CT90, or minimum saturation, nadir, or average) may be stored. Furthermore, all these data can be related to the body position during sleep (whether the subject is supine or in a lateral position, etc.). Conversely, imaging offers anatomical details [87]. Together, they can give a full picture of the severity and potential causes of OSA [88,89]. PSG can help determine the necessity and urgency of treatment, while imaging can guide the selection and planning of specific interventions, such as by determining the suitability for oral appliance therapy or surgical options [16,28,40]. Following treatment, PSG can assess the improvement in sleep-related parameters [90], while imaging can visualize changes in airway structure, helping to evaluate the overall success of the treatment [16,18]. The combination of PSG and imaging facilitates a multidisciplinary approach to OSA management involving sleep physicians, radiologists, ENT specialists, and dental professionals.

### 3.10. What Is the Potential for New Imaging Technologies in the Diagnosis and Management of OSA?

**Figure 5:**
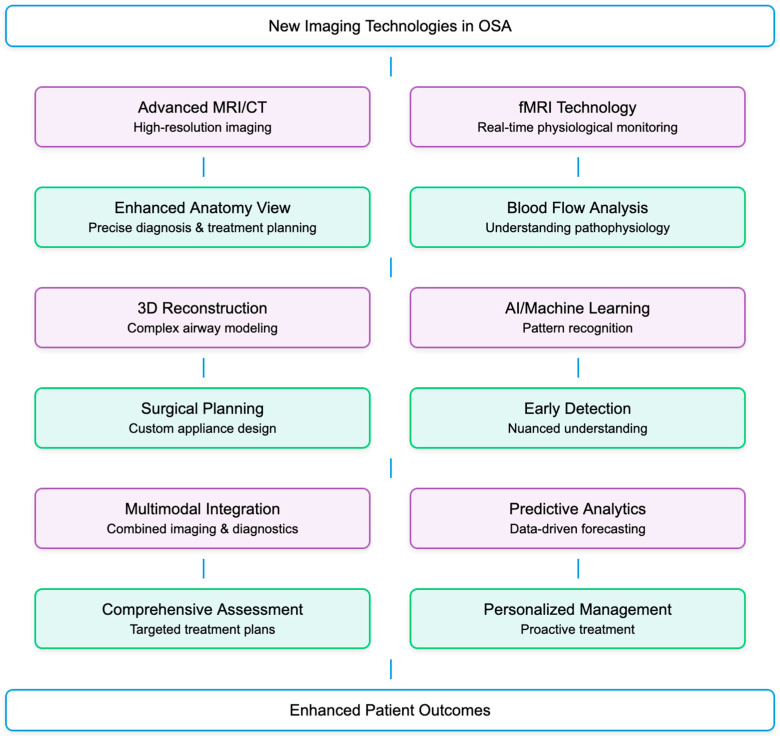
Flow diagram on advancements and innovative techniques in OSA imaging.

The potential for new imaging technologies in the diagnosis and management of OSA is substantial. Innovations in imaging technology continue to enhance our understanding of OSA and improve the precision with which we can identify and treat this condition. Advances in MRI and CT scan technology provide high-resolution images that offer detailed views of the airway anatomy. This can improve the accuracy of OSA diagnosis and help in identifying the most appropriate treatment approach [12,17,32,91,92]. In addition, techniques like fMRI can provide insights into the physiological processes during sleep, such as changes in blood flow, which may contribute to the pathophysiology of OSA [36,93,94]. Three-Dimensional Reconstruction is a 3D imaging technique that allows for a better understanding of the complex structures within the airway and can be used to create models for surgical planning or to design custom-fitted oral appliances. [33,38,95,96,97]. AI and machine learning algorithms can analyze vast amounts of imaging data to identify patterns that may not be apparent to the human eye. This can lead to earlier detection and a more nuanced understanding of OSA [67,98,99] (Figure 5).

Combining imaging data with information from other diagnostic tools, such as polysomnography, can lead to a more comprehensive assessment of OSA, facilitating a more targeted and effective treatment plan [100,101,102,103].

Imaging data, when combined with predictive analytics, could help anticipate the progression of OSA and the response to different treatments, allowing for more proactive and personalized management of the condition [26,32,34,35,101].

## 4. Limitations

While this narrative review provides a comprehensive synthesis of the current advancements in imaging techniques and their application in OSA, it is not without limitations. One of the primary constraints is the exclusion of articles that were not available for free as the full text, which may have led to the omission of relevant data and findings. Additionally, this review is based predominantly on studies available in English, potentially excluding valuable research published in other languages. Furthermore, the inherent nature of a narrative review, as opposed to a systematic review, may introduce selection bias, as the included studies were chosen based on relevance rather than through a pre-defined systematic approach.

## 5. Conclusions

The integration of advanced imaging technologies has the transformative potential to revolutionize the diagnosis and personalized treatment of OSA, offering unprecedented insights into the complex interplay of anatomical, physiological, and functional factors underlying the disorder. High-resolution imaging modalities, complemented by dynamic imaging techniques and AI-driven analytics, empower clinicians with a comprehensive understanding of OSA pathophysiology, enabling the customization of therapeutic interventions tailored to individual patient profiles. As these innovative technologies continue to evolve, they hold promise for not only diagnosing OSA but also predicting treatment responses and long-term outcomes, thereby facilitating proactive and personalized patient management strategies. However, the successful implementation of these cutting-edge technologies in clinical practice necessitates addressing multifaceted challenges, including financial considerations, equitable access to advanced diagnostic tools, and the imperative for standardized protocols across healthcare settings. Moreover, ethical considerations, such as safeguarding patient privacy and ensuring data security, require meticulous attention as these innovations evolve. Sustained interdisciplinary research endeavors and collaborative partnerships are indispensable to harness the full potential of these groundbreaking technologies, ensuring that they translate into tangible improvements in patient care, treatment outcomes, and quality of life for individuals affected by OSA.

## Figures and Tables

**Table 1:** Comparison of imaging procedures for OSA evaluation.

Imaging Procedures	Advantages	Disadvantages
Polysomnography [10,19,22]	Gold standard for OSA diagnosis; measures multiple physiological parameters during sleep.	Expensive; requires overnight stay; limited anatomical detail.
MRI [17,31,32]	Excellent soft tissue contrast; no ionizing radiation; detailed anatomical assessment.	Costly; time-consuming; may be contraindicated for patients with metal implants; may be uncomfortable due to noise and confinement.
CT [25,33]	High-resolution images; good for bony structures; faster than MRI.	Exposure to ionizing radiation; less soft tissue contrast compared to MRI.
CBCT [14]	Lower radiation dose compared to conventional CT; detailed 3D reconstructions of the airway; good for bone assessment.	Limited soft tissue contrast; still involves ionizing radiation.
Ultrasound (PoCUS) [13]	Portable; non-invasive; no ionizing radiation; can be used bedside.	Operator-dependent; limited penetration and resolution; less effective for airway assessment.
Cephalometry [34,35]	Standardized; good for skeletal and dental analysis; low radiation dose.	Two-dimensional limitation; limited to craniofacial structure assessment.
fMRI [36]	Functional assessment of airway during different phases of respiration; no ionizing radiation.	Limited availability; expensive; lower spatial resolution compared to MRI.
3D Airway Reconstruction [37,38]	Detailed visualization of airway anatomy; useful for surgical planning.	May require software for reconstruction; dependent on the quality of the source images.

## Data Availability

All the data reported are present on the PubMed web database.

## Abbreviations

OSA (Obstructive Sleep Apnea), MRI (Magnetic Resonance Imaging), CT (Computed Tomography), CBCT (Cone-Beam Computed Tomography), PoCUS (Point-of-Care Ultrasound), DISE (Drug-Induced Sleep Endoscopy), PSG (Polysomnography), AHI (Apnea Hypopnea Index), AI (Artificial Intelligence), fMRI (Functional Magnetic Resonance Imaging), CPAP (Continuous Positive Airway Pressure), MADs (Mandibular Advancement Devices), UARS (Upper Airway Resistance Syndrome), AHI (Apnea–Hypopnea Index), RDI (Respiratory Disturbance Index), SDB (Sleep-Disordered Breathing), MMA (Maxillomandibular Advancement), UPPP (Uvulopalatopharyngoplasty), Convolutional neural networks (CNNs).
